# Supplementary material for: Evaluation of Machine Learning Interatomic Potentials for Gold Nanoparticles—Transferability towards Bulk
Source: Nanomaterials (Basel). 2023 Jun 9;13(12):1832. doi: 10.3390/nano13121832 (PMC10303715; doi:10.3390/nano13121832)
Supplement: Supplementary file 1 [file nanomaterials-13-01832-s001.zip › nanomaterials-2395324-supplementary.pdf]

Supplementary Material

EVALUATION OF MACHINE LEARNING INTERATOMIC POTENTIALS - TRANSFERABILITY TOWARDS BULK

Marco Fronzi [1], Roger D. Amos [1] and Rika Kobayashi [2]

[1] University of Technology Sydney
[2] Australian National University

Table S1: Geometry of Au_{30} as optimised by VASP
Table S2: Geometry of Au_{30} as optimised by LAMMPS
Table S3: Geometry of Au_{55} (decahedral) as optimised by VASP
Table S4: Geometry of Au_{55} (decahedral) as optimised by LAMMPS
Table S5: Geometry of Au_{55} (cube) as optimised by VASP
Table S6: Geometry of Au_{55} (cube) as optimised by LAMMPS
Table S7: Geometry of Au_{55} (icosahedral) as optimised by VASP
Table S8: Geometry of Au_{55} (icosahedral) as optimised by LAMMPS
Table S9: Geometry of Au_{58} optimised by VASP
Table S10: Geometry of Au_{58} optimised by LAMMPS
Table S11: Geometry of Au_{147} (decahedron) optimised with VASP
Table S12: Geometry of Au_{147} (decahedron) optimised with LAMMPS
Table S13: Geometry of Au_{147} (cube) optimised with VASP
Table S14: Geometry of Au_{147} (cube) optimised with LAMMPS
Table S15: Geometry of Au_{147} (icosahedral) optimised with VASP
Table S16: Geometry of Au_{147} (icosahedral) optimised with LAMMPS
Table S17: Geometry of Au_{147} (amorphous) optimised with VASP
Table S18: Geometry of Au_{147} (amorphous) optimised with LAMMPS
All coordinates are in Ångstrom

Figure S1: Comparison of bond lengths of cube and decahedron versions of Au_{55}
Figure S2: Comparison of bond lengths of cube and decahedron versions of Au_{147}

Au	4.281934000	0.383198000	2.376898000
Au	-1.761646000	3.759514000	0.000181000
Au	-0.929099000	-3.030114000	-0.000185000
Au	1.881397000	-2.704437000	0.000042000
Au	-0.098433000	2.070949000	1.545959000
Au	1.549335000	0.100181000	2.572085000
Au	2.668361000	2.436043000	-1.414929000
Au	-0.098438000	2.071173000	-1.546002000
Au	-4.497160000	3.228104000	0.000182000
Au	0.458877000	-2.532235000	-2.441453000
Au	-1.235688000	-0.284481000	2.593317000
Au	1.549247000	0.100404000	-2.572751000
Au	-2.867053000	1.707878000	1.635848000
Au	-3.965774000	-0.713172000	-2.395234000
Au	2.668500000	2.436319000	1.415315000
Au	0.458890000	-2.532380000	2.441188000
Au	1.000016000	4.175283000	0.000153000
Au	4.281969000	0.382983000	-2.376271000
Au	-2.279865000	-2.889335000	-2.425698000
Au	4.168895000	-1.118294000	0.000317000
Au	-3.593857000	-2.212505000	-0.000160000
Au	-1.235713000	-0.283969000	-2.593908000
Au	-2.279859000	-2.889886000	2.425344000
Au	3.183871000	-2.162471000	-2.439547000
Au	4.966246000	1.620763000	0.000110000
Au	-2.867036000	1.708060000	-1.635657000
Au	-3.965684000	-0.713588000	2.395060000
Au	-4.792136000	0.391758000	0.000022000
Au	3.183946000	-2.162550000	2.440049000
Au	0.165956000	-0.343196000	-0.000275000

Table S1: Geometry of Au_{30} as optimised by VASP

E=-77.933 eV

Au	19.3866	15.5249	17.2887
Au	13.0913	18.9108	15.0016
Au	14.0521	11.6193	14.996
Au	17.1128	11.966	14.9916
Au	14.872	17.2593	16.4807
Au	16.6476	14.9668	17.8238
Au	17.6635	17.4588	13.6005
Au	14.8724	17.2619	13.512
Au	10.3548	18.229	15.0004
Au	15.5145	12.3979	12.667
Au	13.7368	14.5964	17.8382
Au	16.6504	14.9648	12.1851
Au	12.1023	16.7593	16.4975
Au	10.9264	14.4378	12.7011
Au	17.6629	17.4608	16.3976
Au	15.5137	12.3889	17.3352
Au	16.1086	19.2958	14.9981
Au	19.3888	15.5208	12.7093
Au	12.506	12.1166	12.7221
Au	19.4976	13.8599	14.9997
Au	11.0163	12.8821	15.001
Au	13.7413	14.6022	12.1714
Au	12.5025	12.1157	17.2793
Au	18.4609	12.8534	12.6645
Au	20.0607	16.8481	15.0007
Au	12.1042	16.7576	13.5047
Au	10.9283	14.439	17.295
Au	9.7571	15.5602	15
Au	18.4642	12.8558	17.3371
Au	15.3035	14.09	14.9998

Table S2: Geometry of Au_{30} as optimised by LAMMPS

E=-79.460 eV

Au	15.00189	15.00097	14.98072
Au	14.99694	17.89673	14.98148
Au	15.00029	16.41108	17.44631
Au	12.66710	16.41009	15.75924
Au	13.55579	16.41300	13.00405
Au	16.44336	16.41819	13.00398
Au	17.33253	16.41468	15.75577
Au	14.99722	12.10312	14.98179
Au	15.00004	13.58913	17.44610
Au	12.66722	13.58985	15.75915
Au	13.55772	13.58324	13.00453
Au	16.44223	13.58347	13.00445
Au	17.33289	13.58713	15.75511
Au	17.45524	15.00317	18.38472
Au	12.54487	14.99993	18.38637
Au	11.03343	14.99984	13.72867
Au	15.00495	14.99931	10.80665
Au	18.96641	14.99963	13.72874
Au	15.00013	14.99992	19.91821
Au	10.31695	14.99992	16.54422
Au	12.10011	14.99910	11.02874
Au	17.90182	14.99992	11.03120
Au	19.68226	14.99964	16.54497
Au	14.99961	20.69284	14.99016
Au	15.00018	19.39833	17.47907
Au	12.64156	19.40163	15.77798
Au	13.53454	19.41374	12.97765
Au	16.46536	19.41384	12.97780
Au	17.35741	19.40235	15.77855
Au	15.00027	17.79093	19.79158
Au	10.44574	17.78962	16.49059
Au	12.18353	17.79328	11.11539
Au	17.81634	17.79368	11.11499
Au	19.55466	17.78966	16.49053
Au	17.35997	17.88573	18.25497
Au	12.64225	17.88648	18.25509
Au	11.19211	17.89837	13.76836
Au	14.99780	17.89340	10.97880
Au	18.80838	17.89870	13.76817
Au	14.99961	9.30653	14.99028
Au	15.00014	10.60152	17.47903
Au	12.64158	10.59819	15.77797
Au	13.53428	10.58506	12.97767
Au	16.46540	10.58598	12.97792
Au	17.35737	10.59754	15.77842
Au	15.00023	12.20895	19.79152
Au	10.44580	12.21025	16.49057
Au	12.18367	12.20627	11.11530
Au	17.81626	12.20640	11.11532
Au	19.55468	12.21017	16.49028
Au	17.35815	12.11936	18.25120
Au	12.64223	12.11336	18.25507
Au	11.19210	12.10152	13.76836
Au	14.99920	12.10409	10.97814
Au	18.80821	12.10115	13.76811

Table S3: Geometry of Au_{55} (decahedral) as optimised by VASP

E=-147.487 eV

Au	15.0	15	15
Au	15.0	17.8975	15
Au	15.0	16.4063	17.4535
Au	12.6666	16.4063	15.7582
Au	13.5579	16.4063	13.0151
Au	16.4421	16.4063	13.0151
Au	17.3334	16.4063	15.7582
Au	15.0	12.1025	15
Au	15.0	13.5937	17.4535
Au	12.6666	13.5937	15.7582
Au	13.5579	13.5937	13.0151
Au	16.4421	13.5937	13.0151
Au	17.3334	13.5937	15.7582
Au	17.4444	15	18.3644
Au	12.5556	15	18.3644
Au	11.0449	15	13.7149
Au	15.0	15	10.8414
Au	18.9551	15	13.7149
Au	15.0	15	19.9262
Au	10.3149	15	16.5223
Au	12.1044	15	11.0146
Au	17.8956	15	11.0146
Au	19.6851	15	16.5223
Au	15.0	20.7037	15
Au	15.0	19.4052	17.4831
Au	12.6385	19.4052	15.7673
Au	13.5405	19.4052	12.9912
Au	16.4595	19.4052	12.9912
Au	17.3615	19.4052	15.7673
Au	15.0	17.772	19.7954
Au	10.4393	17.772	16.4819
Au	12.1813	17.772	11.1204
Au	17.8187	17.772	11.1204
Au	19.5607	17.772	16.4819
Au	17.349	17.8895	18.2331
Au	12.651	17.8895	18.2331
Au	11.1993	17.8895	13.7651
Au	15.0	17.8895	11.0037
Au	18.8007	17.8895	13.7651
Au	15.0	9.29626	15
Au	15.0	10.5948	17.4831
Au	12.6385	10.5948	15.7673
Au	13.5405	10.5948	12.9912
Au	16.4595	10.5948	12.9912
Au	17.3615	10.5948	15.7673
Au	15.0	12.228	19.7954
Au	10.4393	12.228	16.4819
Au	12.1813	12.228	11.1204
Au	17.8187	12.228	11.1204
Au	19.5607	12.228	16.4819
Au	17.349	12.1105	18.2331
Au	12.651	12.1105	18.2331
Au	11.1993	12.1105	13.7651
Au	15.0	12.1105	11.0037
Au	18.8007	12.1105	13.7651

Table S4: Geometry of Au_{55} (decahedral) as optimised by LAMMPS

E=-146.062 eV

Au	15.00000	15.00000	15.00000
Au	17.01785	17.04679	15.00000
Au	15.00000	17.04679	17.01785
Au	12.98215	17.04679	15.00000
Au	15.00000	17.04679	12.98215
Au	17.02125	15.00000	17.02125
Au	12.97875	15.00000	17.02125
Au	12.97875	15.00000	12.97875
Au	17.02125	15.00000	12.97875
Au	12.98215	12.95321	15.00000
Au	15.00000	12.95321	12.98215
Au	17.01785	12.95321	15.00000
Au	15.00000	12.95321	17.01785
Au	15.00000	19.22001	15.00000
Au	15.00000	15.00000	19.14723
Au	19.14723	15.00000	15.00000
Au	15.00000	10.77999	15.00000
Au	15.00000	15.00000	10.85277
Au	10.85277	15.00000	15.00000
Au	17.03876	19.12354	17.03876
Au	17.02752	17.03570	19.09168
Au	19.09168	17.03570	17.02752
Au	12.96124	10.87646	12.96124
Au	12.97248	12.96430	10.90832
Au	10.90832	12.96430	12.97248
Au	12.96124	19.12354	17.03876
Au	17.02752	12.96430	19.09168
Au	19.09168	17.03570	12.97248
Au	17.03876	10.87646	12.96124
Au	12.97248	17.03570	10.90832
Au	10.90832	12.96430	17.02752
Au	17.03876	19.12354	12.96124
Au	12.97248	17.03570	19.09168
Au	19.09168	12.96430	17.02752
Au	12.96124	10.87646	17.03876
Au	17.02752	12.96430	10.90832
Au	10.90832	17.03570	12.97248
Au	12.96124	19.12354	12.96124
Au	12.97248	12.96430	19.09168
Au	19.09168	12.96430	12.97248
Au	17.03876	10.87646	17.03876
Au	17.02752	17.03570	10.90832
Au	10.90832	17.03570	17.02752
Au	18.95163	18.96131	15.00000
Au	15.00000	18.96131	18.95163
Au	11.04837	18.96131	15.00000
Au	15.00000	18.96131	11.04837
Au	18.94453	15.00000	18.94453
Au	11.05547	15.00000	18.94453
Au	11.05547	15.00000	11.05547
Au	18.94453	15.00000	11.05547
Au	11.04837	11.03869	15.00000
Au	15.00000	11.03869	11.04837
Au	18.95163	11.03869	15.00000
Au	15.00000	11.03869	18.95163

Table S5: Geometry of Au_{55} (cube) as optimised by VASP

E=-148.172 eV

Au	15.0	15	15
Au	17.0196	17.0196	15
Au	15.0	17.0196	17.0196
Au	12.9804	17.0196	15
Au	15.0	17.0196	12.9804
Au	17.0196	15	17.0196
Au	12.9804	15	17.0196
Au	12.9804	15	12.9804
Au	17.0196	15	12.9804
Au	12.9804	12.9804	15
Au	15.0	12.9804	12.9804
Au	17.0196	12.9804	15
Au	15.0	12.9804	17.0196
Au	15.0	19.203	15
Au	15.0	15	19.203
Au	19.203	15	15
Au	15.0	10.797	15
Au	15.0	15	10.797
Au	10.797	15	15
Au	17.0231	19.0992	17.0231
Au	17.0231	17.0231	19.0992
Au	19.0992	17.0231	17.0231
Au	12.9769	10.9008	12.9769
Au	12.9769	12.9769	10.9008
Au	10.9008	12.9769	12.9769
Au	12.9769	19.0992	17.0231
Au	17.0231	12.9769	19.0992
Au	19.0992	17.0231	12.9769
Au	17.0231	10.9008	12.9769
Au	12.9769	17.0231	10.9008
Au	10.9008	12.9769	17.0231
Au	17.0231	19.0992	12.9769
Au	12.9769	17.0231	19.0992
Au	19.0992	12.9769	17.0231
Au	12.9769	10.9008	17.0231
Au	17.0231	12.9769	10.9008
Au	10.9008	17.0231	12.9769
Au	12.9769	19.0992	12.9769
Au	12.9769	12.9769	19.0992
Au	19.0992	12.9769	12.9769
Au	17.0231	10.9008	17.0231
Au	17.0231	17.0231	10.9008
Au	10.9008	17.0231	17.0231
Au	18.945	18.945	15
Au	15.0	18.945	18.945
Au	11.055	18.945	15
Au	15.0	18.945	11.055
Au	18.945	15	18.945
Au	11.055	15	18.945
Au	11.055	15	11.055
Au	18.945	15	11.055
Au	11.055	11.055	15
Au	15.0	11.055	11.055
Au	18.945	11.055	15
Au	15.0	11.055	18.945

Table S6: Geometry of Au_{55} (cube) as optimised by LAMMPS

E=-148.182 eV

Au	15.00000	15.00000	15.00000
Au	15.00000	17.76798	14.98205
Au	17.34915	16.23638	15.76461
Au	16.45800	16.23886	12.99418
Au	13.54200	16.23886	12.99418
Au	12.65085	16.23638	15.76461
Au	15.00000	16.23495	17.46952
Au	15.00000	13.76505	12.53048
Au	12.65085	13.76362	14.23539
Au	13.54200	13.76114	17.00582
Au	16.45800	13.76114	17.00582
Au	17.34915	13.76362	14.23539
Au	15.00000	12.23202	15.01795
Au	15.00000	20.46334	14.99415
Au	15.00000	17.44725	19.87998
Au	10.36068	17.44170	16.52296
Au	12.12630	17.45460	11.04334
Au	17.87370	17.45460	11.04334
Au	19.63932	17.44170	16.52296
Au	15.00000	19.10464	17.53112
Au	12.58285	19.10057	15.76171
Au	13.50598	19.11646	12.93686
Au	16.49402	19.11646	12.93686
Au	17.41715	19.10057	15.76171
Au	12.58564	17.53649	18.32122
Au	11.08797	17.53624	13.72880
Au	15.00000	17.53653	10.89346
Au	18.91203	17.53624	13.72880
Au	17.41436	17.53649	18.32122
Au	16.49138	14.99941	19.59374
Au	11.09374	14.99351	17.84013
Au	11.09374	15.00649	12.15987
Au	16.49138	15.00059	10.40626
Au	19.81163	15.00000	15.00000
Au	15.00000	9.53666	15.00585
Au	15.00000	12.55275	10.12002
Au	19.63932	12.55830	13.47704
Au	17.87370	12.54540	18.95666
Au	12.12630	12.54540	18.95666
Au	10.36068	12.55830	13.47704
Au	15.00000	10.89536	12.46888
Au	17.41715	10.89943	14.23829
Au	16.49402	10.88354	17.06314
Au	13.50598	10.88354	17.06314
Au	12.58285	10.89943	14.23829
Au	17.41436	12.46351	11.67878
Au	18.91203	12.46376	16.27120
Au	15.00000	12.46347	19.10654
Au	11.08797	12.46376	16.27120
Au	12.58564	12.46351	11.67878
Au	13.50862	15.00059	10.40626
Au	18.90626	15.00649	12.15987
Au	18.90626	14.99351	17.84013
Au	13.50862	14.99941	19.59374
Au	10.18837	15.00000	15.00000

Table S7: Geometry of Au_{55} (icosahedral) as optimised by VASP

E=-148.680 eV

Au	15.0	15	15
Au	15.0	17.7612	15
Au	17.3488	16.2348	15.7632
Au	16.4516	16.2348	13.002
Au	13.5483	16.2348	13.002
Au	12.6512	16.2348	15.7632
Au	15.0	16.2348	17.4697
Au	15.0	13.7652	12.5303
Au	12.6512	13.7652	14.2368
Au	13.5484	13.7652	16.998
Au	16.4517	13.7652	16.998
Au	17.3488	13.7652	14.2368
Au	15.0	12.2388	15
Au	15.0	20.4601	15
Au	15.0	17.4418	19.8836
Au	10.3554	17.4418	16.5091
Au	12.1295	17.4418	11.0491
Au	17.8705	17.4418	11.0491
Au	19.6446	17.4418	16.5091
Au	15.0	19.103	17.5358
Au	12.5883	19.103	15.7836
Au	13.5095	19.103	12.9485
Au	16.4905	19.103	12.9485
Au	17.4117	19.103	15.7836
Au	12.5883	17.5358	18.3194
Au	11.0978	17.5358	13.7321
Au	15.0	17.5358	10.897
Au	18.9022	17.5358	13.7321
Au	17.4117	17.5358	18.3194
Au	16.4905	15	19.5873
Au	11.0978	15	17.8351
Au	11.0978	15	12.1649
Au	16.4905	15	10.4127
Au	19.8233	15	15
Au	15.0	9.53993	15
Au	15.0	12.5582	10.1164
Au	19.6446	12.5582	13.4909
Au	17.8705	12.5582	18.9509
Au	12.1295	12.5582	18.9509
Au	10.3554	12.5582	13.4909
Au	15.0	10.897	12.4642
Au	17.4117	10.897	14.2164
Au	16.4905	10.897	17.0515
Au	13.5095	10.897	17.0515
Au	12.5883	10.897	14.2164
Au	17.4117	12.4642	11.6806
Au	18.9022	12.4642	16.2679
Au	15.0	12.4642	19.103
Au	11.0978	12.4642	16.2679
Au	12.5883	12.4642	11.6806
Au	13.5095	15	10.4127
Au	18.9022	15	12.1649
Au	18.9022	15	17.8351
Au	13.5095	15	19.5873
Au	10.1767	15	15

Table S8: Geometry of Au_{55} (icosahedral) as optimised by LAMMPS

E=-148.361 eV

Au	14.56306	14.97117	15.04267
Au	14.64512	14.96389	17.72339
Au	15.68650	12.68904	16.41762
Au	12.27590	12.69543	16.35592
Au	12.24417	17.32816	16.40343
Au	15.58711	17.30438	16.46834
Au	17.22677	15.02599	16.46237
Au	17.21784	15.03762	13.56289
Au	15.55503	17.30731	13.68217
Au	12.26077	17.33765	13.59679
Au	12.28468	12.71554	13.57062
Au	15.65399	12.71292	13.62846
Au	14.61031	15.00232	12.35474
Au	15.83277	12.60593	19.36595
Au	13.06758	13.14522	19.13035
Au	13.08825	16.80630	19.13843
Au	15.83851	17.31517	19.39772
Au	17.46777	14.96964	19.41652
Au	18.41184	12.60223	18.25934
Au	16.63679	10.43357	17.82917
Au	13.93492	10.92390	17.74079
Au	11.61472	14.99061	17.71263
Au	13.91252	19.07016	17.80454
Au	16.62120	19.53803	17.95100
Au	18.41367	17.36411	18.30550
Au	19.79993	15.00392	17.77980
Au	18.22069	12.87728	15.00994
Au	16.53558	10.23414	15.01021
Au	13.78854	10.79733	14.96236
Au	11.55184	15.02706	14.97092
Au	13.73094	19.26836	15.02463
Au	16.47938	19.77375	15.12304
Au	18.11651	17.21881	15.03343
Au	20.95832	15.01637	15.05604
Au	19.85100	15.04738	12.33543
Au	18.45989	17.42758	11.86265
Au	16.66566	19.56515	12.30719
Au	13.95834	19.10949	12.25106
Au	11.61542	15.02608	12.22427
Au	13.98624	10.97854	12.18830
Au	16.68568	10.49387	12.20080
Au	18.45729	12.66978	11.81156
Au	17.53618	15.06167	10.65551
Au	15.90214	17.40882	10.74105
Au	13.13624	16.87055	10.88104
Au	13.12032	13.21393	10.81108
Au	15.90029	12.70144	10.68554
Au	14.92407	15.07069	9.59194
Au	20.66448	12.74166	16.56523
Au	20.57017	17.30868	16.48631
Au	20.69590	12.75498	13.52881
Au	20.61915	17.34357	13.64871
Au	18.97198	10.44838	16.46999
Au	18.99605	19.57044	13.73812
Au	19.00682	10.46663	13.56652
Au	18.94793	19.52898	16.52526
Au	14.83392	14.95653	20.47801
Au	21.32681	10.54566	15.04899

Table S9: Geometry of Au_{58} optimised by VASP

E=-159.119 eV

Au	14.5292	15.0002	15.0222
Au	14.5855	14.9688	17.7786
Au	15.6519	12.7321	16.4178
Au	12.6404	12.8547	16.3867
Au	12.6225	17.1182	16.4291
Au	15.617	17.2506	16.4652
Au	17.27	15.0029	16.5347
Au	17.2901	15.0345	13.5471
Au	15.636	17.2804	13.6419
Au	12.6413	17.1477	13.6348
Au	12.6593	12.8843	13.5874
Au	15.6709	12.762	13.5943
Au	14.6226	15.0271	12.2668
Au	15.7788	12.6363	19.3184
Au	13.0432	13.0391	19.1928
Au	13.0536	16.8815	19.2206
Au	15.7894	17.3076	19.3558
Au	17.3022	14.9814	19.3963
Au	18.2951	12.641	18.1286
Au	16.6638	10.4051	17.7751
Au	13.9815	10.8612	17.7735
Au	11.6222	14.9705	17.8034
Au	13.9492	19.1025	17.8521
Au	16.6338	19.5466	17.852
Au	18.3172	17.3434	18.1595
Au	19.7851	14.9924	17.8566
Au	18.1581	12.8434	15.0238
Au	16.6451	10.2678	14.9864
Au	13.944	10.8192	14.9741
Au	11.5621	14.9965	15.0022
Au	13.9129	19.1964	15.0624
Au	16.5874	19.7981	15.0867
Au	18.1217	17.206	15.0697
Au	20.1333	15.0158	15.0601
Au	19.8228	15.0516	12.2592
Au	18.3587	17.4086	11.9863
Au	16.6711	19.605	12.3176
Au	13.9867	19.1614	12.2721
Au	11.6599	15.0296	12.2021
Au	14.0191	10.9203	12.1769
Au	16.7013	10.464	12.2018
Au	18.3369	12.7067	11.9176
Au	17.3608	15.0734	10.6864
Au	15.8472	17.3985	10.7556
Au	13.11	16.97	10.845
Au	13.0997	13.1279	10.7915
Au	15.8368	12.7274	10.6942
Au	14.7211	15.0755	9.4994
Au	20.5963	12.7219	16.5625
Au	20.5575	17.3316	16.52
Au	20.6168	12.7541	13.5159
Au	20.5768	17.3619	13.6552
Au	19.0232	10.3814	16.4737
Au	19.0295	19.6356	13.7123
Au	19.043	10.4125	13.5341
Au	19.0108	19.6062	16.4902
Au	14.6468	14.9587	20.5475
Au	21.3163	10.5847	15.0212

Table S10: Geometry of Au_{58} optimised by LAMMPS

E=-158.893 eV

Au	15.00057	14.99691	14.98108
Au	15.00115	17.88278	15.00360
Au	15.00002	16.42263	17.48608
Au	12.60922	16.42305	15.75908
Au	13.52895	16.42110	12.97418
Au	16.47087	16.42257	12.97348
Au	17.39134	16.42559	15.75720
Au	15.00567	12.11623	15.00508
Au	15.00018	13.57709	17.48699
Au	12.60812	13.57536	15.75866
Au	13.52892	13.57690	12.97482
Au	16.47168	13.57744	12.97343
Au	17.39290	13.57732	15.75889
Au	17.42890	15.00062	18.33488
Au	12.57052	14.99989	18.33437
Au	11.04495	14.99427	13.71610
Au	14.99684	14.99979	10.85905
Au	18.95503	15.00254	13.71547
Au	14.99455	15.00039	19.96860
Au	10.26194	15.00256	16.53686
Au	12.07067	14.99707	10.96915
Au	17.92946	14.99974	10.96565
Au	19.73926	15.00769	16.53542
Au	14.98975	20.73859	15.01365
Au	14.99908	19.29962	17.48547
Au	12.62762	19.30019	15.77148
Au	13.54347	19.29411	12.99251
Au	16.45014	19.30608	12.98803
Au	17.36542	19.30671	15.76172
Au	15.00123	17.80885	19.93368
Au	10.28851	17.81238	16.52959
Au	12.10497	17.81122	11.00455
Au	17.89534	17.81264	11.00492
Au	19.70363	17.82633	16.51880
Au	17.39968	17.85107	18.29282
Au	12.60069	17.85245	18.29620
Au	11.11400	17.83984	13.73936
Au	15.00023	17.84547	10.91466
Au	18.88130	17.85295	13.73247
Au	14.98877	9.25849	15.01865
Au	14.99866	10.70020	17.48604
Au	12.63015	10.70033	15.77287
Au	13.54646	10.70457	12.99332
Au	16.45501	10.69778	12.99859
Au	17.36977	10.69777	15.77304
Au	15.00344	12.19014	19.93513
Au	10.28693	12.18957	16.52892
Au	12.10487	12.18595	11.00459
Au	17.89480	12.18432	11.00667
Au	19.71097	12.19233	16.52831
Au	17.39919	12.15012	18.29356
Au	12.60061	12.14787	18.29631
Au	11.11268	12.14701	13.73730
Au	15.00055	12.15390	10.91506
Au	18.88471	12.14865	13.73751
Au	14.99783	23.53118	15.01372
Au	14.99579	6.46455	15.01461
Au	17.38292	22.20336	15.78444
Au	15.00013	22.20886	17.51832
Au	12.61209	22.20110	15.78623
Au	13.52650	22.20368	12.98815
Au	16.47130	22.20946	12.99255
Au	17.38290	7.79943	15.78420
Au	15.00046	7.79041	17.51866

Au	12.61167	7.79861	15.78654
Au	13.52143	7.79930	12.98950
Au	16.46054	7.78957	12.99189
Au	19.69059	20.76146	16.52444
Au	15.00070	20.75670	19.91660
Au	10.30756	20.75480	16.52583
Au	12.11882	20.76929	11.02896
Au	17.88553	20.78245	11.03054
Au	19.69147	9.24104	16.52332
Au	15.00073	9.24263	19.91617
Au	10.30761	9.24533	16.52573
Au	12.11897	9.23071	11.02809
Au	17.88192	9.22201	11.03335
Au	18.92689	20.92912	13.73776
Au	17.42740	20.93163	18.33448
Au	12.57306	20.93121	18.33581
Au	11.07530	20.92441	13.73694
Au	15.00083	20.93955	10.88277
Au	18.92248	9.07008	13.73850
Au	17.42787	9.06808	18.33447
Au	12.57302	9.06852	18.33568
Au	11.07441	9.07538	13.73911
Au	15.00220	9.05682	10.87912
Au	21.18816	19.28654	14.46765
Au	17.41222	19.30667	20.69590
Au	10.30945	19.30577	19.05630
Au	9.71029	19.30105	11.79411
Au	16.41211	19.30793	8.97353
Au	21.18726	10.71324	14.46836
Au	17.41162	10.69309	20.69628
Au	10.30972	10.69437	19.05557
Au	9.71040	10.68822	11.79131
Au	16.41225	10.69252	8.97306
Au	19.69619	19.30552	19.05096
Au	12.60118	19.31271	20.70282
Au	8.81761	19.28583	14.46892
Au	13.58847	19.30747	8.97274
Au	20.29197	19.30762	11.79290
Au	19.69472	10.69545	19.05015
Au	12.59989	10.68785	20.70260
Au	8.81047	10.71346	14.47046
Au	13.58868	10.69273	8.97256
Au	20.29132	10.69156	11.79341
Au	21.93549	19.19091	17.24904
Au	15.02237	19.19402	22.27247
Au	8.06442	19.18659	17.26245
Au	10.72146	19.20058	9.12206
Au	19.27755	19.20095	9.12237
Au	21.93345	10.81220	17.24898
Au	15.01407	10.79947	22.27079
Au	8.06435	10.81316	17.26272
Au	10.72151	10.79925	9.12018
Au	19.27842	10.79896	9.12288
Au	17.51357	16.41764	20.86853
Au	10.19332	16.42003	19.19687
Au	9.50178	16.42043	11.72240
Au	16.41846	16.41730	8.75337
Au	21.39478	16.41628	14.41688
Au	17.51354	13.58215	20.86856
Au	10.19306	13.57982	19.19657
Au	9.50119	13.57692	11.72232
Au	16.41879	13.58236	8.75375
Au	21.39387	13.58381	14.42108
Au	12.48267	16.41947	20.86721

Au	8.60614	16.41566	14.42142
Au	13.58164	16.41731	8.75316
Au	20.49796	16.42114	11.72123
Au	19.80646	16.42075	19.19482
Au	12.48317	13.58024	20.86720
Au	8.60558	13.58358	14.42139
Au	13.58170	13.58240	8.75307
Au	20.49872	13.57694	11.72287
Au	19.80660	13.58092	19.19685
Au	15.00015	16.38605	22.39823
Au	7.95187	16.38165	17.29394
Au	10.63778	16.39108	9.02199
Au	19.36223	16.39491	9.01865
Au	22.05097	16.38856	17.28236
Au	14.99982	13.61104	22.39966
Au	7.95183	13.61853	17.29399
Au	10.63662	13.60845	9.02157
Au	19.36600	13.61073	9.02334
Au	22.04737	13.62042	17.29288

Table S11: Geometry of Au_{147} (decahedron) optimised with VASP

E=-418.248 eV

Au	15.0	15	15
Au	15.0	17.9554	15
Au	15.0	16.4484	17.4598
Au	12.6606	16.4484	15.7601
Au	13.5542	16.4484	13.01
Au	16.4458	16.4484	13.01
Au	17.3394	16.4484	15.7601
Au	15.0	12.0446	15
Au	15.0	13.5516	17.4598
Au	12.6606	13.5516	15.7601
Au	13.5542	13.5516	13.01
Au	16.4458	13.5516	13.01
Au	17.3394	13.5516	15.7601
Au	17.4186	15	18.3289
Au	12.5814	15	18.3289
Au	11.0867	15	13.7285
Au	15.0	15	10.8853
Au	18.9133	15	13.7285
Au	15.0	15	19.935
Au	10.3065	15	16.525
Au	12.0993	15	11.0075
Au	17.9007	15	11.0075
Au	19.6935	15	16.525
Au	15.0	20.8349	15
Au	15.0	19.3667	17.4697
Au	12.6512	19.3667	15.7632
Au	13.5484	19.3667	13.002
Au	16.4516	19.3667	13.002
Au	17.3488	19.3667	15.7632
Au	15.0	17.8219	19.8947
Au	10.3449	17.8219	16.5126
Au	12.123	17.8219	11.0401
Au	17.877	17.8219	11.0401
Au	19.6551	17.8219	16.5125
Au	17.378	17.8894	18.273
Au	12.622	17.8894	18.273
Au	11.1523	17.8894	13.7498
Au	15.0	17.8894	10.9543
Au	18.8477	17.8894	13.7498
Au	15.0	9.16514	15
Au	15.0	10.6333	17.4697
Au	12.6512	10.6333	15.7632

Au	13.5484	10.6333	13.002
Au	16.4516	10.6333	13.002
Au	17.3488	10.6333	15.7632
Au	15.0	12.1781	19.8947
Au	10.3449	12.1781	16.5126
Au	12.123	12.1781	11.0401
Au	17.877	12.1781	11.0401
Au	19.6551	12.1781	16.5125
Au	17.378	12.1106	18.273
Au	12.622	12.1106	18.273
Au	11.1523	12.1106	13.7498
Au	15.0	12.1106	10.9543
Au	18.8477	12.1106	13.7498
Au	15.0	23.6259	15
Au	15.0	6.37406	15
Au	17.3909	22.2676	15.7768
Au	15.0	22.2676	17.5139
Au	12.6091	22.2676	15.7769
Au	13.5223	22.2676	12.9662
Au	16.4777	22.2676	12.9662
Au	17.3909	7.7324	15.7768
Au	15.0	7.7324	17.5139
Au	12.6091	7.7324	15.7769
Au	13.5223	7.7324	12.9662
Au	16.4777	7.7324	12.9662
Au	19.6848	20.8005	16.5222
Au	15.0	20.8005	19.9259
Au	10.3152	20.8005	16.5222
Au	12.1046	20.8005	11.0149
Au	17.8954	20.8005	11.0149
Au	19.6848	9.19947	16.5222
Au	15.0	9.19947	19.9259
Au	10.3152	9.19947	16.5222
Au	12.1046	9.19947	11.0149
Au	17.8954	9.19947	11.0149
Au	18.9179	20.9378	13.727
Au	17.4214	20.9378	18.3328
Au	12.5786	20.9378	18.3328
Au	11.0821	20.9378	13.727
Au	15.0	20.9378	10.8804
Au	18.9179	9.06221	13.727
Au	17.4214	9.06221	18.3328
Au	12.5786	9.06221	18.3328
Au	11.0821	9.06221	13.727
Au	15.0	9.06221	10.8804
Au	21.1667	19.2946	14.4822
Au	17.3981	19.2946	20.7049
Au	10.3154	19.2946	19.0436
Au	9.70664	19.2946	11.7942
Au	16.4131	19.2946	8.97507
Au	21.1667	10.7054	14.4822
Au	17.3981	10.7054	20.7049
Au	10.3154	10.7054	19.0436
Au	9.70664	10.7054	11.7942
Au	16.4131	10.7054	8.97507
Au	19.6846	19.2946	19.0436
Au	12.6019	19.2946	20.7049
Au	8.83326	19.2946	14.4822
Au	13.5868	19.2946	8.97507
Au	20.2934	19.2946	11.7942
Au	19.6846	10.7054	19.0436
Au	12.6019	10.7054	20.7049
Au	8.83326	10.7054	14.4822
Au	13.5868	10.7054	8.97507

Au	20.2934	10.7054	11.7942
Au	21.8977	19.1455	17.2412
Au	15.0	19.1455	22.2527
Au	8.10226	19.1455	17.2412
Au	10.737	19.1455	9.13243
Au	19.263	19.1455	9.13242
Au	21.8977	10.8545	17.2412
Au	15.0	10.8545	22.2527
Au	8.10226	10.8545	17.2412
Au	10.737	10.8545	9.13243
Au	19.263	10.8545	9.13242
Au	17.5032	16.4201	20.8579
Au	10.2024	16.4201	19.1909
Au	9.53169	16.4201	11.7323
Au	16.418	16.4201	8.78954
Au	21.3447	16.4201	14.4295
Au	17.5032	13.5799	20.8579
Au	10.2024	13.5799	19.1909
Au	9.53169	13.5799	11.7323
Au	16.418	13.5799	8.78954
Au	21.3447	13.5799	14.4295
Au	12.4968	16.4201	20.8579
Au	8.65531	16.4201	14.4295
Au	13.582	16.4201	8.78954
Au	20.4683	16.4201	11.7322
Au	19.7976	16.4201	19.1909
Au	12.4968	13.5799	20.8579
Au	8.65531	13.5799	14.4295
Au	13.582	13.5799	8.78954
Au	20.4683	13.5799	11.7322
Au	19.7976	13.5799	19.1909
Au	15.0	16.3745	22.3737
Au	7.98721	16.3745	17.2786
Au	10.6659	16.3745	9.03457
Au	19.3341	16.3745	9.03456
Au	22.0128	16.3745	17.2786
Au	15.0	13.6255	22.3737
Au	7.98721	13.6255	17.2786
Au	10.6659	13.6255	9.03457
Au	19.3341	13.6255	9.03456
Au	22.0128	13.6255	17.2786

Table S12: Geometry of Au_{147} (decahedron) optimised with LAMMPS

E=-419.082 eV

Au	15.00000	15.00000	15.00000
Au	17.04295	17.04805	14.99679
Au	15.00326	17.04811	17.04270
Au	12.96099	17.04736	15.00080
Au	14.99924	17.04720	12.95989
Au	17.03826	15.00004	17.03776
Au	12.96114	15.00035	17.03941
Au	12.96174	14.99996	12.96224
Au	17.03886	14.99965	12.96059
Au	12.95705	12.95195	15.00321
Au	14.99674	12.95189	12.95730
Au	17.03901	12.95264	14.99920
Au	15.00075	12.95280	17.04011
Au	15.00475	19.19712	15.00081
Au	14.99643	14.99880	19.19386
Au	19.19355	15.00000	14.99515
Au	14.99525	10.80288	14.99919
Au	15.00357	15.00120	10.80614
Au	10.80645	15.00000	15.00485
Au	17.02706	19.10675	17.02680
Au	17.02569	17.02600	19.09466
Au	19.09340	17.02772	17.02415
Au	12.97294	10.89325	12.97320
Au	12.97431	12.97400	10.90534
Au	10.90660	12.97228	12.97585
Au	12.97403	19.10655	17.02629
Au	17.02530	12.97288	19.09460
Au	19.09447	17.02847	12.97428
Au	17.02597	10.89345	12.97371
Au	12.97470	17.02712	10.90540
Au	10.90553	12.97153	17.02572
Au	17.02595	19.10785	12.97215
Au	12.97450	17.02763	19.09465
Au	19.09417	12.97252	17.02450
Au	12.97405	10.89215	17.02785
Au	17.02550	12.97237	10.90535
Au	10.90583	17.02748	12.97550
Au	12.97390	19.10664	12.97384
Au	12.97454	12.97169	19.09427
Au	19.09411	12.97182	12.97433
Au	17.02610	10.89336	17.02616
Au	17.02546	17.02831	10.90573
Au	10.90589	17.02818	17.02567
Au	19.04625	19.05241	15.00059
Au	15.00099	19.05197	19.04616
Au	10.95481	19.05149	14.99778
Au	14.99709	19.05095	10.95362
Au	19.04456	14.99828	19.04230
Au	10.95491	14.99857	19.04294
Au	10.95544	15.00172	10.95770
Au	19.04509	15.00143	10.95706
Au	10.95375	10.94759	14.99941
Au	14.99901	10.94803	10.95384
Au	19.04519	10.94851	15.00222
Au	15.00291	10.94905	19.04638
Au	17.01136	21.30319	14.99997
Au	14.99898	17.01301	21.29111
Au	21.28605	15.00005	17.00597
Au	12.98864	8.69681	15.00004
Au	15.00102	12.98699	8.70889
Au	8.71395	14.99995	12.99403
Au	14.99995	21.30291	17.01072
Au	17.01256	14.99871	21.29056
Au	21.29108	17.01738	14.99881

Au	15.00005	8.69709	12.98928
Au	12.98744	15.00129	8.70944
Au	8.70892	12.98262	15.00119
Au	12.98946	21.30259	15.00002
Au	14.99906	12.98338	21.29104
Au	21.29166	15.00134	12.98583
Au	17.01054	8.69741	14.99998
Au	15.00094	17.01662	8.70896
Au	8.70834	14.99866	17.01417
Au	14.99999	21.30285	12.98928
Au	12.98390	14.99930	21.29073
Au	21.28988	12.98400	14.99734
Au	15.00001	8.69715	17.01072
Au	17.01610	15.00070	8.70927
Au	8.71012	17.01600	15.00266
Au	19.02703	21.11051	17.05177
Au	17.05321	19.02349	21.10000
Au	21.09865	17.05487	19.02269
Au	10.97297	8.88949	12.94823
Au	12.94679	10.97651	8.90000
Au	8.90135	12.94513	10.97731
Au	17.05172	21.11050	19.02701
Au	19.02377	17.05309	21.10349
Au	21.10404	19.02552	17.05266
Au	12.94828	8.88950	10.97299
Au	10.97623	12.94691	8.89651
Au	8.89596	10.97448	12.94734
Au	17.04964	21.11012	10.97016
Au	10.97610	17.05468	21.10343
Au	21.10373	10.97555	17.05448
Au	12.95036	8.88988	19.02984
Au	19.02390	12.94532	8.89657
Au	8.89627	19.02445	12.94552
Au	19.02608	21.10959	12.94726
Au	12.94706	19.02499	21.10395
Au	21.10287	12.94621	19.02351
Au	10.97392	8.89041	17.05274
Au	17.05294	10.97501	8.89605
Au	8.89713	17.05379	10.97649
Au	10.97354	21.10958	12.94765
Au	12.94737	10.97451	21.10423
Au	21.10313	12.94579	10.97593
Au	19.02646	8.89042	17.05235
Au	17.05263	19.02549	8.89577
Au	8.89687	17.05421	19.02407
Au	12.94842	21.11038	10.97284
Au	10.97611	12.94306	21.10335
Au	21.10244	10.97469	12.94485
Au	17.05158	8.88962	19.02716
Au	19.02389	17.05694	8.89665
Au	8.89756	19.02531	17.05515
Au	12.94851	21.11019	19.02707
Au	19.02419	12.94613	21.10366
Au	21.10412	19.02596	12.94745
Au	17.05149	8.88981	10.97293
Au	10.97581	17.05387	8.89634
Au	8.89588	10.97404	17.05255
Au	10.97365	21.10933	17.05212
Au	17.05626	10.97545	21.10387
Au	21.10141	17.05910	10.97597
Au	19.02635	8.89067	12.94788
Au	12.94374	19.02455	8.89613
Au	8.89859	12.94090	19.02403
Au	19.20645	19.20958	19.20571

Au	10.79432	19.20928	19.20672
Au	19.20604	10.79068	19.20651
Au	19.20555	19.20995	10.79352
Au	10.79355	10.79042	10.79429
Au	19.20568	10.79072	10.79328
Au	10.79396	19.20932	10.79349
Au	10.79445	10.79005	19.20648
Au	20.97945	20.96876	14.99994
Au	15.00002	20.96684	20.98083
Au	9.01881	20.96502	15.00003
Au	14.99929	20.96551	9.01830
Au	20.96390	15.00007	20.96380
Au	9.03630	14.99962	20.96368
Au	9.03610	14.99993	9.03620
Au	20.96370	15.00038	9.03632
Au	9.02055	9.03124	15.00006
Au	14.99998	9.03316	9.01917
Au	20.98119	9.03498	14.99997
Au	15.00071	9.03449	20.98170

Table S13: Geometry of Au_{147} (cube) optimised with VASP

E=-417.731 eV

Au	15.0	15	15
Au	17.0442	17.0442	15
Au	15.0	17.0442	17.0442
Au	12.9558	17.0442	15
Au	15.0	17.0442	12.9558
Au	17.0442	15	17.0442
Au	12.9558	15	17.0442
Au	12.9558	15	12.9558
Au	17.0442	15	12.9558
Au	12.9558	12.9558	15
Au	15.0	12.9558	12.9558
Au	17.0442	12.9558	15
Au	15.0	12.9558	17.0442
Au	15.0	19.1703	15
Au	15.0	15	19.1703
Au	19.1703	15	15
Au	15.0	10.8297	15
Au	15.0	15	10.8297
Au	10.8297	15	15
Au	17.0293	19.1081	17.0293
Au	17.0293	17.0293	19.1081
Au	19.1081	17.0293	17.0293
Au	12.9707	10.8919	12.9707
Au	12.9707	12.9707	10.8919
Au	10.8919	12.9707	12.9707
Au	12.9707	19.108	17.0293
Au	17.0293	12.9707	19.108
Au	19.108	17.0293	12.9707
Au	17.0293	10.892	12.9707
Au	12.9707	17.0293	10.892
Au	10.892	12.9707	17.0293
Au	17.0293	19.1081	12.9707
Au	12.9707	17.0293	19.1081
Au	19.1081	12.9707	17.0293
Au	12.9707	10.8919	17.0293
Au	17.0293	12.9707	10.8919
Au	10.8919	17.0293	12.9707
Au	12.9707	19.1081	12.9707
Au	12.9707	12.9707	19.1081
Au	19.1081	12.9707	12.9707
Au	17.0293	10.8919	17.0293
Au	17.0293	17.0293	10.8919

Au	10.8919	17.0293	17.0293
Au	19.0257	19.0258	15
Au	15.0	19.0257	19.0258
Au	10.9742	19.0257	15.0001
Au	14.9999	19.0258	10.9743
Au	19.0258	15	19.0257
Au	10.9743	14.9999	19.0258
Au	10.9742	15	10.9743
Au	19.0257	15.0001	10.9742
Au	10.9743	10.9742	15
Au	15.0	10.9743	10.9742
Au	19.0258	10.9743	14.9999
Au	15.0001	10.9742	19.0257
Au	17.0021	21.2955	15
Au	15.0	17.0021	21.2955
Au	21.2955	15	17.0021
Au	12.9979	8.70452	15
Au	15.0	12.9979	8.70452
Au	8.70452	15	12.9979
Au	15.0	21.2955	17.0022
Au	17.0022	15	21.2955
Au	21.2955	17.0022	15
Au	15.0	8.70455	12.9978
Au	12.9978	15	8.70455
Au	8.70455	12.9978	15
Au	12.9978	21.2955	15.0001
Au	15.0001	12.9978	21.2955
Au	21.2955	15.0001	12.9978
Au	17.0022	8.70455	14.9999
Au	14.9999	17.0022	8.70455
Au	8.70455	14.9999	17.0022
Au	14.9999	21.2955	12.9979
Au	12.9979	14.9999	21.2955
Au	21.2955	12.9979	14.9999
Au	15.0001	8.70452	17.0021
Au	17.0021	15.0001	8.70452
Au	8.70452	17.0021	15.0001
Au	19.0034	21.1304	17.0375
Au	17.0375	19.0034	21.1304
Au	21.1304	17.0375	19.0034
Au	10.9966	8.86965	12.9625
Au	12.9625	10.9966	8.86965
Au	8.86965	12.9625	10.9966
Au	17.0374	21.1303	19.0035
Au	19.0035	17.0374	21.1303
Au	21.1303	19.0035	17.0374
Au	12.9626	8.86967	10.9965
Au	10.9965	12.9626	8.86967
Au	8.86967	10.9965	12.9626
Au	17.0374	21.1304	10.9966
Au	10.9966	17.0374	21.1304
Au	21.1304	10.9966	17.0374
Au	12.9626	8.86962	19.0034
Au	19.0034	12.9626	8.86962
Au	8.86962	19.0034	12.9626
Au	19.0034	21.1304	12.9626
Au	12.9626	19.0034	21.1304
Au	21.1304	12.9626	19.0034
Au	10.9966	8.86962	17.0374
Au	17.0374	10.9966	8.86962
Au	8.86962	17.0374	10.9966
Au	10.9965	21.1303	12.9626
Au	12.9626	10.9965	21.1303
Au	21.1303	12.9626	10.9965

Au	19.0035	8.86967	17.0374
Au	17.0374	19.0035	8.86967
Au	8.86967	17.0374	19.0035
Au	12.9625	21.1304	10.9966
Au	10.9966	12.9625	21.1304
Au	21.1304	10.9966	12.9625
Au	17.0375	8.86965	19.0034
Au	19.0034	17.0375	8.86965
Au	8.86965	19.0034	17.0375
Au	12.9625	21.1303	19.0035
Au	19.0035	12.9625	21.1303
Au	21.1303	19.0035	12.9625
Au	17.0375	8.8697	10.9965
Au	10.9965	17.0375	8.8697
Au	8.8697	10.9965	17.0375
Au	10.9965	21.1303	17.0375
Au	17.0375	10.9965	21.1303
Au	21.1303	17.0375	10.9965
Au	19.0035	8.8697	12.9625
Au	12.9625	19.0035	8.8697
Au	8.8697	12.9625	19.0035
Au	19.1492	19.1492	19.1492
Au	10.8508	19.1491	19.1492
Au	19.1492	10.8508	19.1491
Au	19.1491	19.1492	10.8508
Au	10.8508	10.8508	10.8508
Au	19.1492	10.8509	10.8508
Au	10.8508	19.1492	10.8509
Au	10.8509	10.8508	19.1492
Au	20.9436	20.9437	15
Au	15.0	20.9436	20.9437
Au	9.05633	20.9436	15.0001
Au	14.9999	20.9437	9.05642
Au	20.9437	15	20.9436
Au	9.05642	14.9999	20.9437
Au	9.05633	15	9.05642
Au	20.9436	15.0001	9.05633
Au	9.05641	9.05633	15
Au	15.0	9.05642	9.05633
Au	20.9437	9.05641	14.9999
Au	15.0001	9.05633	20.9436

Table S14: Geometry of Au_{147} (cube) optimised with LAMMPS

E=-418.787 eV

Au	14.98805	14.99934	14.98751
Au	14.99998	17.77762	15.00117
Au	17.35677	16.23887	15.76779
Au	16.46052	16.23669	12.98835
Au	13.53913	16.24366	12.98358
Au	12.63468	16.24563	15.76700
Au	14.99986	16.23662	17.47651
Au	14.99790	13.75681	12.51116
Au	12.63117	13.75581	14.23025
Au	13.53914	13.76259	17.00789
Au	16.45740	13.76166	17.00539
Au	17.35740	13.75239	14.23087
Au	14.99845	12.22256	14.99451
Au	15.00266	20.52785	15.01313
Au	15.00271	17.46802	19.93848
Au	10.28923	17.46631	16.53172
Au	12.10029	17.46518	10.98941
Au	17.90307	17.46224	10.99343
Au	19.70796	17.46307	16.53365
Au	15.00136	19.06676	17.52428
Au	12.59600	19.07469	15.76796
Au	13.52489	19.07684	12.96662
Au	16.47805	19.07486	12.96531
Au	17.40476	19.07148	15.76604
Au	12.60126	17.52005	18.29686
Au	11.12871	17.52021	13.73527
Au	15.00546	17.53347	10.92791
Au	18.87289	17.51241	13.73730
Au	17.39922	17.51946	18.29636
Au	16.47798	14.99701	19.55720
Au	11.12252	15.00276	17.81699
Au	11.12292	14.99917	12.18487
Au	16.47737	14.99612	10.44094
Au	19.78463	14.99992	15.00371
Au	15.00260	9.47600	14.98729
Au	15.00203	12.52073	10.05614
Au	19.71005	12.53579	13.46906
Au	17.90195	12.53774	19.00433
Au	12.09850	12.53785	19.00681
Au	10.28727	12.53494	13.47041
Au	15.00191	10.93050	12.47264
Au	17.40614	10.92533	14.23173
Au	16.47791	10.92850	17.03434
Au	13.52221	10.92830	17.03469
Au	12.59648	10.92699	14.23403
Au	17.40080	12.47927	11.70301
Au	18.87370	12.48698	16.26393
Au	14.99925	12.46896	19.06823
Au	11.12529	12.48266	16.26486
Au	12.60010	12.47963	11.70319
Au	13.52252	14.99712	10.44069
Au	18.87755	14.99680	12.18503
Au	18.87699	15.00254	17.81709
Au	13.52190	15.00168	19.55643
Au	10.20838	15.00424	15.00458
Au	15.00030	23.23520	15.00080
Au	15.00025	18.68253	22.36227
Au	7.98721	18.69106	17.27113
Au	10.66950	18.68460	9.04313
Au	19.33022	18.68420	9.04465
Au	22.01134	18.69063	17.27194
Au	17.46895	20.50791	18.40060
Au	12.53155	20.50832	18.40134
Au	10.99675	20.51661	13.70197

Au	15.00232	20.51880	10.78885
Au	19.00649	20.51497	13.69744
Au	14.99988	13.70291	21.79684
Au	8.52565	13.69580	17.10906
Au	10.99403	13.69951	9.48694
Au	19.00622	13.69944	9.48684
Au	21.47369	13.69428	17.10784
Au	14.99522	21.87164	17.56771
Au	12.55852	21.87183	15.79504
Au	13.50221	21.87390	12.93055
Au	16.51107	21.87273	12.93223
Au	17.44087	21.87030	15.79690
Au	12.55714	18.78616	20.79676
Au	8.73798	18.78584	14.47073
Au	13.56275	18.78810	8.87808
Au	20.37662	18.77887	11.74698
Au	19.76337	18.78245	19.11051
Au	17.44526	18.78588	20.79428
Au	10.23773	18.78232	19.11055
Au	9.63757	18.80096	11.74656
Au	16.43986	18.78878	8.87670
Au	21.26152	18.78580	14.47189
Au	15.00011	20.36347	20.00996
Au	10.23760	20.36288	16.54704
Au	12.06051	20.36562	10.96058
Au	17.94462	20.36142	10.95945
Au	19.76183	20.36182	16.54553
Au	13.49824	16.22528	22.07381
Au	7.79823	16.22004	15.75301
Au	12.05133	16.20683	8.38429
Au	20.37841	16.21799	10.15955
Au	21.27483	16.21637	18.61771
Au	16.50455	16.22540	22.07413
Au	8.72521	16.21663	18.61735
Au	9.62194	16.21888	10.16022
Au	17.94877	16.20683	8.38450
Au	22.19510	16.21697	15.75714
Au	15.00007	6.77609	14.99940
Au	15.00012	11.30622	7.63137
Au	22.01434	11.30998	12.73018
Au	19.33053	11.31680	20.95602
Au	10.67138	11.31949	20.95863
Au	7.98557	11.30966	12.73013
Au	12.52823	9.49519	11.60185
Au	17.46942	9.49206	11.59932
Au	19.00383	9.48521	16.29831
Au	15.00001	9.48112	19.20949
Au	10.99539	9.48606	16.30210
Au	15.00006	16.29629	8.20340
Au	21.47630	16.30415	12.89320
Au	19.00437	16.29949	20.51382
Au	10.99377	16.30416	20.51433
Au	8.52751	16.30618	12.89352
Au	14.99280	8.12815	12.43071
Au	17.44146	8.12991	14.20261
Au	16.50743	8.12850	17.06954
Au	13.49272	8.12870	17.06955
Au	12.55791	8.12882	14.20593
Au	17.44750	11.21429	9.20539
Au	21.26195	11.21463	15.52942
Au	16.44015	11.21644	21.11757
Au	9.63314	11.20895	18.25831
Au	10.23562	11.22175	10.89361
Au	12.55381	11.21390	9.20532

Au	19.76739	11.21958	10.89034
Au	20.36494	11.20344	18.25502
Au	13.56279	11.21204	21.12160
Au	8.72995	11.21611	15.53402
Au	15.00210	9.63080	9.98709
Au	19.76626	9.63811	13.45383
Au	17.94172	9.63704	19.04223
Au	12.05715	9.63770	19.04346
Au	10.23653	9.64167	13.46503
Au	16.50147	13.76637	7.92422
Au	22.19374	13.78965	14.24469
Au	17.94838	13.78770	21.61877
Au	9.62104	13.78150	19.84233
Au	8.72529	13.78382	11.38342
Au	13.49730	13.76490	7.92599
Au	21.27498	13.78338	11.38269
Au	20.37899	13.78105	19.84218
Au	12.05090	13.79310	21.61555
Au	7.80251	13.78064	14.25484

Table S15: Geometry of Au_{147} (icosahedral) optimised with VASP

E=-420.674 eV

Au	15.0	15	15
Au	15.0	17.8078	15
Au	17.3885	16.2557	15.7761
Au	16.4761	16.2557	12.9683
Au	13.5239	16.2557	12.9683
Au	12.6115	16.2557	15.7761
Au	15.0	16.2557	17.5114
Au	15.0	13.7443	12.4886
Au	12.6115	13.7443	14.2239
Au	13.5239	13.7443	17.0317
Au	16.4761	13.7443	17.0317
Au	17.3885	13.7443	14.2239
Au	15.0	12.1922	15
Au	15.0	20.5463	15
Au	15.0	17.4804	19.9607
Au	10.2821	17.4804	16.533
Au	12.0842	17.4804	10.9867
Au	17.9158	17.4804	10.9867
Au	19.7179	17.4804	16.533
Au	15.0	19.1087	17.5393
Au	12.5849	19.1087	15.7847
Au	13.5074	19.1087	12.9456
Au	16.4926	19.1087	12.9456
Au	17.4151	19.1087	15.7847
Au	12.5849	17.5393	18.324
Au	11.0924	17.5393	13.7303
Au	15.0	17.5393	10.8913
Au	18.9076	17.5393	13.7303
Au	17.4151	17.5393	18.324
Au	16.4926	15	19.5937
Au	11.0924	15	17.8391
Au	11.0924	15	12.1609
Au	16.4926	15	10.4063
Au	19.8301	15	15
Au	15.0	9.45373	15
Au	15.0	12.5196	10.0393
Au	19.7179	12.5196	13.467
Au	17.9158	12.5196	19.0133
Au	12.0842	12.5196	19.0133
Au	10.2821	12.5196	13.467
Au	15.0	10.8913	12.4607
Au	17.4151	10.8913	14.2153

Au	16.4926	10.8913	17.0544
Au	13.5074	10.8913	17.0544
Au	12.5849	10.8913	14.2153
Au	17.4151	12.4607	11.676
Au	18.9076	12.4607	16.2697
Au	15.0	12.4607	19.1087
Au	11.0924	12.4607	16.2697
Au	12.5849	12.4607	11.676
Au	13.5074	15	10.4063
Au	18.9076	15	12.1609
Au	18.9076	15	17.8391
Au	13.5074	15	19.5937
Au	10.1699	15	15
Au	15.0	23.2751	15
Au	15.0	18.7007	22.4014
Au	7.96081	18.7007	17.2872
Au	10.6495	18.7007	9.01211
Au	19.3505	18.7007	9.0121
Au	22.0392	18.7007	17.2872
Au	17.4593	20.477	18.385
Au	12.5407	20.477	18.385
Au	11.0207	20.477	13.7071
Au	15.0	20.477	10.8159
Au	18.9793	20.477	13.7071
Au	15.0	13.7071	21.7699
Au	8.5614	13.7071	17.092
Au	11.0207	13.7071	9.523
Au	18.9793	13.7071	9.523
Au	21.4386	13.7071	17.092
Au	15.0	21.9056	17.5604
Au	12.5649	21.9056	15.7912
Au	13.495	21.9056	12.9286
Au	16.505	21.9056	12.9286
Au	17.4351	21.9056	15.7912
Au	12.5649	18.796	20.8227
Au	8.70978	18.796	14.4834
Au	13.5475	18.796	8.85801
Au	20.3925	18.796	11.7206
Au	19.7852	18.796	19.1152
Au	17.4351	18.796	20.8227
Au	10.2148	18.796	19.1152
Au	9.60746	18.796	11.7206
Au	16.4525	18.796	8.85801
Au	21.2902	18.796	14.4834
Au	15.0	20.3784	20.0315
Au	10.2148	20.3784	16.5548
Au	12.0426	20.3784	10.9294
Au	17.9574	20.3784	10.9294
Au	19.7852	20.3784	16.5548
Au	13.495	16.2355	22.1029
Au	7.77966	16.2355	15.7636
Au	12.0426	16.2355	8.36901
Au	20.3925	16.2355	10.1382
Au	21.2902	16.2355	18.6262
Au	16.505	16.2355	22.1029
Au	8.70978	16.2355	18.6262
Au	9.60746	16.2355	10.1382
Au	17.9574	16.2355	8.36901
Au	22.2203	16.2355	15.7636
Au	15.0	6.72493	15
Au	15.0	11.2993	7.59855
Au	22.0392	11.2993	12.7128
Au	19.3505	11.2993	20.9879
Au	10.6495	11.2993	20.9879

Au	7.96081	11.2993	12.7128
Au	12.5407	9.523	11.615
Au	17.4593	9.523	11.615
Au	18.9793	9.523	16.2929
Au	15.0	9.523	19.1841
Au	11.0207	9.523	16.2929
Au	15.0	16.2929	8.23006
Au	21.4386	16.2929	12.908
Au	18.9793	16.2929	20.477
Au	11.0207	16.2929	20.477
Au	8.5614	16.2929	12.908
Au	15.0	8.0944	12.4396
Au	17.4351	8.0944	14.2088
Au	16.505	8.0944	17.0714
Au	13.495	8.0944	17.0714
Au	12.5649	8.0944	14.2088
Au	17.4351	11.204	9.17728
Au	21.2902	11.204	15.5166
Au	16.4525	11.204	21.142
Au	9.60746	11.204	18.2794
Au	10.2148	11.204	10.8848
Au	12.5649	11.204	9.17728
Au	19.7852	11.204	10.8848
Au	20.3925	11.204	18.2794
Au	13.5475	11.204	21.142
Au	8.70978	11.204	15.5166
Au	15.0	9.62162	9.96849
Au	19.7852	9.62162	13.4452
Au	17.9574	9.62162	19.0706
Au	12.0426	9.62162	19.0706
Au	10.2148	9.62162	13.4452
Au	16.505	13.7645	7.89708
Au	22.2203	13.7645	14.2364
Au	17.9574	13.7645	21.631
Au	9.60746	13.7645	19.8618
Au	8.70978	13.7645	11.3738
Au	13.495	13.7645	7.89708
Au	21.2902	13.7645	11.3738
Au	20.3925	13.7645	19.8618
Au	12.0426	13.7645	21.631
Au	7.77966	13.7645	14.2364

Table S16: Geometry of Au_{147} (icosahedral) optimised with LAMMPS

E=-419.309 eV

Au	7.95069	24.70054	12.97571
Au	8.53579	19.90954	12.63877
Au	9.36314	22.56077	12.04252
Au	5.63070	21.72996	15.99196
Au	8.46201	24.93429	17.49451
Au	4.58143	21.56189	18.57438
Au	16.02848	22.30791	16.89184
Au	10.69130	24.94630	12.94757
Au	12.19916	26.12657	15.10294
Au	6.78467	24.33006	15.37656
Au	13.53162	23.61413	16.40905
Au	6.36202	22.58916	20.47286
Au	8.92765	23.10178	21.75748
Au	9.30257	25.98093	15.10939
Au	6.12027	23.70133	17.96930
Au	9.91627	24.77219	19.75312
Au	7.13742	22.07024	13.65408
Au	12.74991	18.45883	10.54899
Au	6.69974	19.49798	14.77222
Au	8.20767	17.41363	13.81404
Au	6.60726	17.02331	16.19276
Au	11.97729	23.16976	11.27635
Au	12.85016	22.44060	13.89936
Au	10.27414	17.90176	11.81419
Au	15.29657	20.27499	15.13584
Au	8.43649	22.15144	16.19262
Au	13.04353	19.78016	13.15957
Au	8.17452	19.61931	18.80528
Au	9.89934	20.42158	20.90620
Au	10.65452	15.39783	18.13968
Au	10.14006	20.22303	17.04138
Au	5.62818	19.30062	17.37863
Au	5.75555	19.74863	20.24763
Au	6.60853	17.29447	19.12600
Au	12.84391	14.80714	19.69791
Au	16.11958	25.46517	12.55495
Au	15.47750	23.22249	14.22117
Au	13.42128	25.08884	12.83323
Au	12.77950	20.82263	16.27437
Au	10.78537	23.63688	15.54567
Au	16.44205	21.79138	9.97839
Au	11.31490	22.70496	18.05443
Au	8.70177	22.37321	18.96109
Au	9.13790	17.55562	16.97043
Au	10.22498	17.80708	19.81022
Au	11.75352	16.41630	21.74149
Au	9.27103	19.41953	23.52256
Au	13.58076	17.25850	25.91806
Au	11.86046	20.35849	22.92098
Au	18.85881	25.19275	12.42817
Au	10.99618	26.01311	17.53364
Au	12.43928	25.91508	19.92767
Au	17.68795	21.53781	14.42558
Au	13.79245	26.19032	17.55591
Au	11.85270	22.49639	20.79193
Au	10.88656	25.31193	22.1991
Au	13.52540	24.46817	22.04919
Au	15.04872	24.92603	19.77554
Au	7.38407	20.87137	22.27770
Au	11.41089	18.16950	24.58105
Au	11.20490	22.96119	23.46322
Au	11.00753	20.53081	11.27006
Au	9.92693	15.40973	12.89637
Au	11.39962	13.27176	11.90602

Au	8.60977	15.14695	15.37506
Au	10.37926	13.17482	14.46027
Au	7.77315	15.07764	18.06311
Au	9.31341	13.36067	19.77956
Au	14.64136	23.71583	10.78728
Au	13.64626	21.12289	10.44119
Au	11.92078	15.75240	10.74589
Au	10.45376	21.11325	14.36397
Au	12.71194	16.80533	13.11184
Au	15.48563	18.09652	13.23986
Au	10.76640	18.38226	14.71679
Au	11.80399	17.92117	17.47167
Au	13.21816	14.16529	17.07237
Au	13.54680	18.09956	15.38304
Au	8.35859	15.70233	20.75602
Au	7.64298	18.19501	21.59241
Au	10.23834	14.15485	22.29788
Au	13.24284	17.63579	19.80073
Au	18.19265	19.96947	11.08727
Au	17.25308	24.41915	10.25426
Au	19.87440	18.18874	12.27379
Au	14.59143	19.06187	17.76232
Au	16.32993	17.52685	15.87914
Au	20.08897	20.75577	13.22815
Au	12.10511	20.13818	19.18765
Au	16.29021	20.62573	19.21188
Au	15.81242	18.24537	20.60987
Au	17.58638	19.92032	16.74134
Au	13.74464	18.16910	22.58761
Au	15.94023	17.95526	24.48023
Au	9.41668	16.68357	23.20003
Au	14.10085	20.56735	21.09967
Au	18.70173	22.56378	11.68596
Au	19.78866	23.38307	14.28832
Au	14.98515	25.91481	15.08035
Au	15.69732	20.95341	12.52213
Au	18.72027	23.49883	16.92513
Au	16.40485	25.11183	17.38479
Au	14.00271	22.31062	18.87527
Au	16.08473	20.54405	23.27941
Au	17.08342	15.90962	23.00667
Au	17.36142	23.35973	19.38882
Au	15.80218	22.93301	21.64537
Au	13.90584	22.24403	23.60081
Au	13.86103	19.80338	24.88714
Au	17.88882	18.56925	22.56897
Au	12.97980	12.26215	13.92218
Au	9.39973	13.02795	17.02520
Au	11.71896	11.62114	16.28718
Au	15.55278	19.11637	10.30871
Au	13.95835	14.15874	12.11423
Au	14.72198	16.52289	10.87091
Au	13.28245	11.65345	18.60534
Au	15.63646	13.02953	14.10693
Au	11.57920	15.75763	15.49979
Au	15.06939	16.13618	18.12083
Au	10.54138	11.11956	18.68906
Au	15.77527	13.28721	18.20783
Au	11.73140	12.35854	20.80807
Au	13.02823	13.60836	22.86907
Au	14.59450	12.70615	20.70753
Au	17.42771	17.32787	11.33734
Au	16.37541	15.27755	12.81351
Au	17.10128	14.74196	15.74479

Au	19.21794	16.55271	16.56077
Au	14.53524	11.78173	16.19819
Au	18.41378	16.63633	13.96730
Au	14.44029	15.47753	14.95693
Au	17.48134	17.73575	18.46279
Au	17.02059	14.09324	20.69337
Au	18.00509	14.93817	18.35291
Au	14.76308	15.74332	21.43637
Au	11.75781	15.44267	24.42542
Au	15.84397	13.36808	23.06128
Au	18.49470	16.45190	20.71515
Au	17.68547	25.14024	14.92274
Au	20.11995	21.21562	16.03591
Au	20.18148	18.59632	14.97070
Au	19.24820	21.41552	18.75484
Au	17.97418	21.23412	21.34721
Au	19.30574	19.05196	20.27154
Au	20.11523	18.94978	17.69126
Au	14.62408	15.37590	24.38064

Table S17: Geometry of Au_{147} (amorphous) optimised with VASP

Au	8.40189	24.2493	13.9578
Au	8.52922	19.4015	12.5852
Au	9.69512	22.6788	12.2039
Au	5.48646	21.5421	15.5941
Au	8.13531	25.1878	17.5767
Au	4.8621	21.818	18.2476
Au	16.0449	22.0296	16.5997
Au	10.9846	24.9812	12.9828
Au	12.2619	25.9313	15.264
Au	6.82708	26.0347	15.3486
Au	13.6267	23.3608	15.984
Au	7.06967	23.1154	19.2652
Au	7.92333	23.7016	21.8423
Au	9.5409	26.2668	15.3218
Au	5.85234	23.9502	16.9081
Au	9.44702	24.6029	19.8054
Au	5.76586	23.7786	14.1256
Au	13.0755	18.6385	10.1078
Au	6.51536	19.2842	14.6032
Au	7.65473	17.0427	13.7858
Au	6.20661	16.9603	16.1965
Au	12.2425	23.1546	11.1081
Au	12.6882	22.2318	13.6242
Au	10.5358	17.7892	11.4735
Au	14.7353	20.3722	14.6508
Au	7.49257	21.6745	13.6496
Au	12.2145	19.4094	13.1162
Au	9.24692	19.1983	19.2845
Au	9.33035	22.3422	23.8576
Au	11.5305	14.4258	18.2507
Au	10.8956	18.9179	17.0439
Au	5.65337	19.3776	17.2534
Au	6.69695	20.3717	19.6123
Au	6.91141	17.6425	18.9936
Au	14.1859	14.9206	18.9303
Au	16.2658	25.7155	12.8306
Au	15.5052	22.9234	13.9325
Au	13.6757	24.9124	12.9062
Au	12.4001	20.8999	16.1048
Au	10.7714	23.4615	15.2942
Au	17.3988	21.8192	10.3433
Au	11.4279	22.976	17.9162
Au	9.58751	21.8328	19.7584

Au	8.81452	17.2384	16.8778
Au	10.3282	16.6685	19.1793
Au	12.4572	15.9056	20.8518
Au	9.12934	19.5633	23.8952
Au	13.7631	16.8923	25.5612
Au	11.3416	20.9217	24.998
Au	17.859	23.5909	12.3722
Au	10.7957	25.7133	17.6381
Au	12.0477	25.3478	20.1098
Au	17.8476	20.7358	14.9403
Au	13.509	25.4773	17.7875
Au	12.1782	22.5862	20.552
Au	10.5722	24.2025	22.2665
Au	13.2959	24.26	22.5011
Au	14.6913	24.5199	20.0815
Au	7.99637	21.0407	21.8725
Au	11.3113	18.2018	24.8499
Au	11.6105	23.6725	24.7161
Au	10.6336	20.4795	11.0899
Au	9.69035	15.6045	12.8619
Au	11.3548	13.7882	11.7447
Au	8.00305	14.895	15.3496
Au	10.1928	13.4562	14.4197
Au	7.60722	15.0861	18.1857
Au	9.28014	13.5557	19.8003
Au	15.2506	23.4047	11.1654
Au	12.4247	21.1568	9.2397
Au	12.4391	16.0714	10.6425
Au	10.2433	20.9212	14.2483
Au	12.3938	16.1486	13.463
Au	14.5425	17.9958	12.5917
Au	10.1768	18.083	14.4685
Au	12.7621	17.1517	17.9258
Au	13.4704	14.5954	16.1502
Au	13.17	18.157	15.3788
Au	8.26335	15.9022	20.8151
Au	7.83645	18.4192	21.5978
Au	10.3392	14.5639	22.0672
Au	13.9876	18.3716	19.9604
Au	18.7985	19.5363	10.121
Au	15.0577	21.6931	9.02926
Au	19.3652	18.9331	12.7568
Au	14.4433	19.4998	17.4493
Au	16.4921	18.2912	15.2269
Au	20.9733	20.6204	14.1766
Au	12.1195	20.2123	19.0318
Au	16.186	20.6921	19.6428
Au	16.1182	18.9203	21.8056
Au	17.3378	19.6887	17.4099
Au	13.7646	17.7558	22.7396
Au	16.1335	18.0863	24.6618
Au	9.5684	16.9626	23.1061
Au	14.1293	20.875	21.4005
Au	19.5862	21.5356	11.9575
Au	19.48	22.8523	14.4804
Au	14.9944	25.7773	15.271
Au	14.0312	20.859	11.595
Au	18.5565	23.0364	17.187
Au	16.134	24.7189	17.5785
Au	14.1553	22.2306	18.5759
Au	16.0569	20.8073	23.8577
Au	17.836	16.8732	22.6836
Au	17.1405	23.2621	19.6265
Au	15.7258	23.034	22.1025

Au	13.6992	22.0995	24.0401
Au	13.7326	19.5512	24.8659
Au	18.4244	19.3836	23.7476
Au	12.8011	12.9955	13.9026
Au	9.27589	13.2029	17.0358
Au	11.7537	12.1909	16.378
Au	15.9483	19.3159	10.0447
Au	14.1245	14.3585	11.928
Au	15.1547	16.5476	10.3609
Au	13.6129	12.078	18.4455
Au	15.5499	13.2628	14.1239
Au	10.9842	15.8385	15.9152
Au	15.4836	16.6552	17.2082
Au	10.6925	11.4447	18.7587
Au	15.7096	13.1898	17.0716
Au	12.063	12.9728	20.5743
Au	13.6222	13.9308	22.5287
Au	15.3172	12.9714	20.4675
Au	17.5204	17.5	11.3706
Au	16.6069	15.2194	12.4517
Au	17.2463	14.8338	15.5816
Au	18.4564	16.6885	17.0785
Au	14.2342	11.401	15.6604
Au	18.298	16.7003	13.9368
Au	14.9922	16.1403	14.4927
Au	16.7005	17.8353	19.2949
Au	17.5529	14.4827	21.4021
Au	17.1565	14.7436	18.7008
Au	15.3605	16.1123	21.0563
Au	12.0507	15.8097	23.7455
Au	16.1279	13.0313	23.127
Au	18.9242	16.4406	20.0582
Au	17.4623	24.5331	15.1132
Au	20.2116	20.9038	16.7882
Au	19.69	18.5165	15.4892
Au	19.0579	21.2604	19.2769
Au	17.9299	21.4804	21.8604
Au	18.9282	19.0622	21.0381
Au	19.7488	18.6567	18.4592
Au	15.4371	15.5455	23.8332

Table S18: Geometry of Au_{147} (amorphous) optimised with LAMMPS

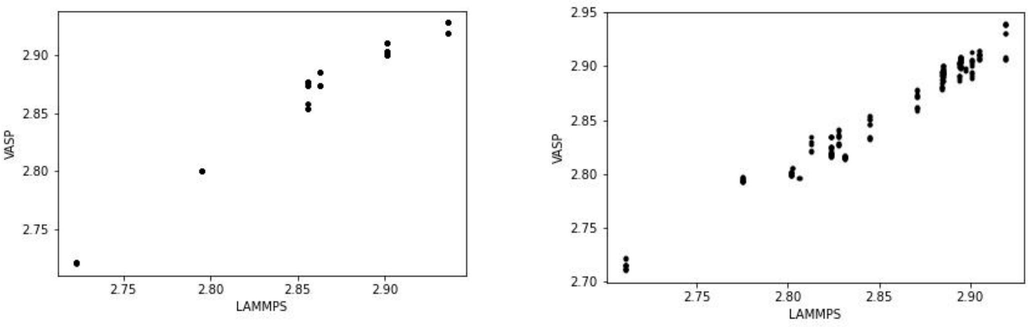

Figure S1: Comparison of bond lengths of cube and decahedron versions of Au_{55}

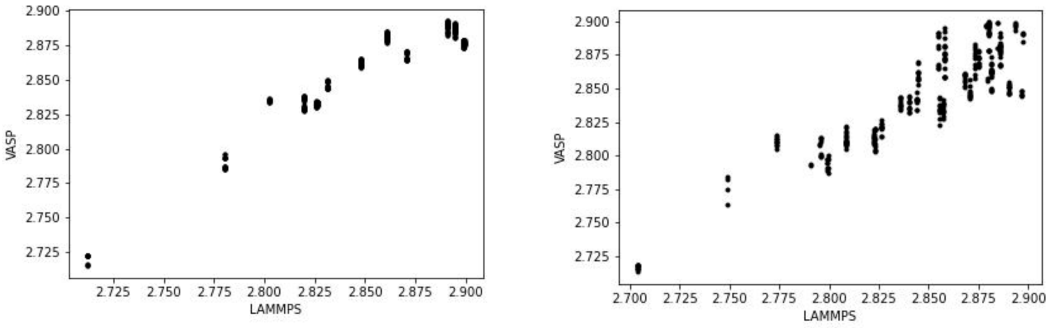

Figure S2: Comparison of bond lengths of cube and decahedron versions of Au_{147}
